# Enzyme‐Linked Cycloaddition Assay (ELCA) for Rapid, Ultra‐Sensitive Monitoring of Secreted Sialoglycoproteins

**DOI:** 10.1002/advs.78315

**Published:** 2026-10-14

**Authors:** Jon Lundstrøm, Jingmei Yang, Daniel Bojar

**Affiliations:** ^1^ Department of Chemistry and Molecular Biology University of Gothenburg Gothenburg Sweden; ^2^ Wallenberg Centre for Molecular and Translational Medicine University of Gothenburg Gothenburg Sweden; ^3^ Science For Life Laboratory (SciLifeLab) University of Gothenburg Gothenburg Sweden

## Abstract

Glycosylation of proteins is central to cell signaling, immune function, and pathogen interactions, yet existing methods for monitoring glycan changes require specialized instrumentation and primarily report on membrane‐anchored, rather than secreted, glycoproteins. Here, we present the Enzyme‐Linked Cycloaddition Assay (ELCA), a click chemistry‐based platform for ultra‐sensitive detection and semi‐quantitative analysis of secreted sialoglycoproteins. By metabolically incorporating an azide‐modified sialic acid into newly synthesized glycoproteins and capturing labeled material via strain‐promoted cycloaddition, ELCA quantifies aggregate sialylation using a microplate reader‐compatible, ELISA‐like workflow. We demonstrate that the secreted glycoproteome responds rapidly to pharmacological perturbation, with changes detectable within hours. Benchmarking against common glycosylation inhibitors and profiling cytokine‐driven macrophage polarization further establishes ELCA's sensitivity and temporal resolution. Compatible with serum‐containing conditions and requiring no specialized instrumentation, ELCA provides a broadly accessible tool for rapid, cost‐effective monitoring of secreted glycoprotein dynamics.

## Graphical abstract

Secreted glycoproteins report on cellular state faster than the cell surface, yet remain hard to measure. The Enzyme‐Linked Cycloaddition Assay (ELCA) combines metabolic azide labeling with click chemistry in an ELISA‐like microplate format, resolving sialylation changes within minutes for 1‐2 EUR per sample. Glycosylation inhibitors and macrophage activation reveal dynamics that surface‐based analyses miss entirely.

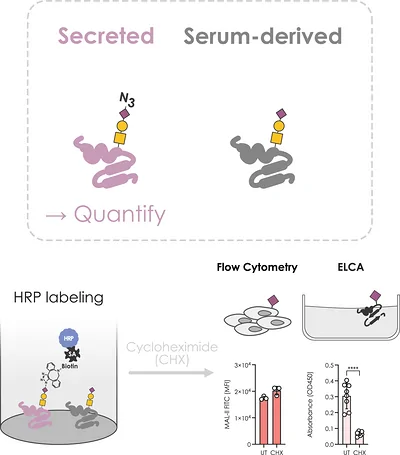

## 1 Introduction

Glycans modify the vast majority of proteins that traverse the secretory pathway, including both membrane‐anchored and secreted proteins [1]. As complex carbohydrate structures exposed to the extracellular environment, glycans play central roles across cell–cell and cell–pathogen interactions [2, 3], tumor immune evasion [4], microbiome development [5], and further regulate the stability, structure, and function of the proteins they modify [6]. Overall, glycosylation is involved in diverse biological processes and is increasingly recognized as an important target in both diagnostic and therapeutic applications [7, 8].

Glycans are enzymatically synthesized in a non‐templated manner, where the availability and flux of protein substrates, glycosyltransferase enzymes, and monosaccharide donors collectively determine the final structures. Investigating the cellular glycome remains inherently challenging, due to extreme structural diversity arising from the assembly of chemically highly similar monosaccharide building blocks [9].

Comprehensive structural characterization using mass spectrometry‐based approaches is powerful but typically expensive, time‐consuming, and dependent on access to specialized instrumentation and expert domain knowledge. Further, representative insights into global motif abundance, such as sialylation, require the acquisition of multiple types of glycomics data (*N*‐, *O*‐glycans, glycosphingolipids) [10, 11]. Alternatively, glycan profiling using recognition by specific glycan‐binding proteins (lectins) as a proxy serves as a viable alternative, as lectin binding patterns can be directly correlated with the glycan motifs present in a sample and often aggregate information from several glycan classes [12], yet suffer from variable specificity of lectins for binding their motif in certain sequence contexts.

Secreted glycoproteins, including cytokines, hormones, growth factors, enzymes, etc., are particularly attractive as a source of material for monitoring cellular activity, allowing for continuous measurements with little to no sample preparation requirements as well as rapid response to changes in the cellular state [13]. In contrast, the glycan profile of membrane‐anchored proteins responds more slowly to perturbations, since pre‐existing surface proteins must first be turned over before changes are observed [14]. However, the analysis of secreted glycoproteins presents several challenges, including relatively low abundances and potential interference from serum‐derived glycoproteins under standard culture conditions, which is why most methods investigate glycosylation levels from the cell lysate (e.g., mass spectrometry) or the membrane itself (e.g., flow cytometry).

Previous studies have developed mass spectrometry‐based protocols for quantifying secreted (glyco)proteins under standard culture conditions, combining bioorthogonal metabolic labeling with specific enrichment and TMT‐based multiplexing to boost signal sensitivity [15, 16]. While such approaches enable detailed protein‐level quantification, they come with the trade‐off of being cost‐ and labor‐intensive, and may not be readily accessible to many laboratories.

Although site‐specific glycosylation can be inferred with such methods, the reported results are typically limited to aggregate *N*‐glycosylation, as quantification relies on the comparison of peptide signals before and after enzyme‐mediated deglycosylation, without providing information on glycan composition. Importantly, it was demonstrated that click chemistry‐based enrichment of secreted glycoproteins alone is insufficient for comprehensive analysis, as non‐specifically retained serum‐derived proteins can still dominate the signal [16].

Several ELISA‐derived methods, such as the Enzyme‐Linked Lectin Assay (ELLA) [17] and Enzyme‐Linked Lectin Binding Assay (ELBA) [18], offer more cost‐effective and accessible approaches for quantifying soluble glycoproteins. However, these methods require serum‐free culture conditions, as glycan motifs indistinguishable from those of the sample of interest are present in highly abundant serum‐derived proteins.

Here, we develop and benchmark a method to detect and quantify rapid changes in the secreted glycoproteome. While higher‐resolution approaches (e.g., LC‐MS/MS‐based glycomics and/or glycoproteomics) can provide detailed structural information, they require substantially more complex experimental setups, as well as time and resource investments. In contrast, our herein developed Enzyme‐Linked Cycloaddition Assay (ELCA) enables monitoring global changes in secreted glycoproteins (total sialylation, i.e., change in total glycoprotein and/or change in glycosylation) using a simple and cost‐effective workflow using click chemistry. The assay is comparable to a standard ELISA setup and requires only access to a microplate reader, thereby facilitating higher‐throughput analyses across replicates, time points, and treatment conditions than existing methods and opening up sensitive glycan analyses to all molecular biology laboratories. Importantly, the per‐sample cost is as low as 1–2 EUR, making it orders of magnitude less expensive than mass spectrometry‐based analyses.

Using ELCA, we demonstrate here that changes in the secreted glycome are detectable at substantially earlier time points compared to analyses of cell surface‐associated glycoproteins. We evaluate the effects of common glycosylation inhibitors and observe rapid impacts on secreted glycoproteins, while conventional cell surface analysis by flow cytometry requires considerably longer treatment durations to reveal comparable changes, providing caveats and guidelines to current usage of such inhibitors in scientific studies. Finally, we characterize the secreted glycoprotein response of THP‐1‐derived macrophages following cytokine‐mediated activation, yielding insights into the early response of glycoprotein secretion. Overall, we envision ELCA as a general platform for assessing the impact of treatments on glycosylation in a rapid, cost‐effective, and sensitive manner.

## 2 Results

### 2.1 ELCA Enables Semi‐Quantitative Analysis of Secreted Glycoproteins Under Standard Culture Conditions

Sialylation, capping glycans with one or multiple sialic acids, is one of the most prominent and relevant glycan features in mammals and thus highly studied for many biological questions. It has been shown to control the half‐life of secreted proteins [19], as well as mediate interactions with immune system proteins such as Siglecs [20, 21] and, being tied to the same biosynthetic machinery as membrane‐associated glycoproteins, is a proxy for general mature cellular glycosylation [22]. We thus aimed to develop a method that would allow any researcher to quantify time‐resolved changes in sialylation sensitively and cost‐effectively that did not require specialized instrumentation. To achieve this aim, such a method had to overcome several hurdles, such as the presence of similar, or even identical, glycans on FBS‐derived glycoproteins in cell culture, or the slow time scales of plasma membrane turnover. Additionally, we caution that, even using mass spectrometry, researchers usually focus on only one type of glycosylation, such as *N*‐ or *O*‐glycomics, and do not necessarily observe changes in the total glycome of a cell.

Taken all these considerations together, we decided to use metabolic labeling to introduce azide groups into sialic acids (Neu5Az, instead of Neu5Ac) that we could then use for bioorthogonal click chemistry to introduce biotin tags that would allow for sensitive detection of secreted sialoglycoproteins, without interference from non‐azide‐containing serum glycoproteins, in a method we termed Enzyme‐Linked Cycloaddition Assay (ELCA, Figure 1A). We note that, thanks to the incorporation of Neu5Az into all glycan classes [23], ELCA thus reported on global sialoglycoprotein levels, combining signals from both *N*‐ and *O*‐glycans, as well as all sialic acid linkages.

**FIGURE 1:**
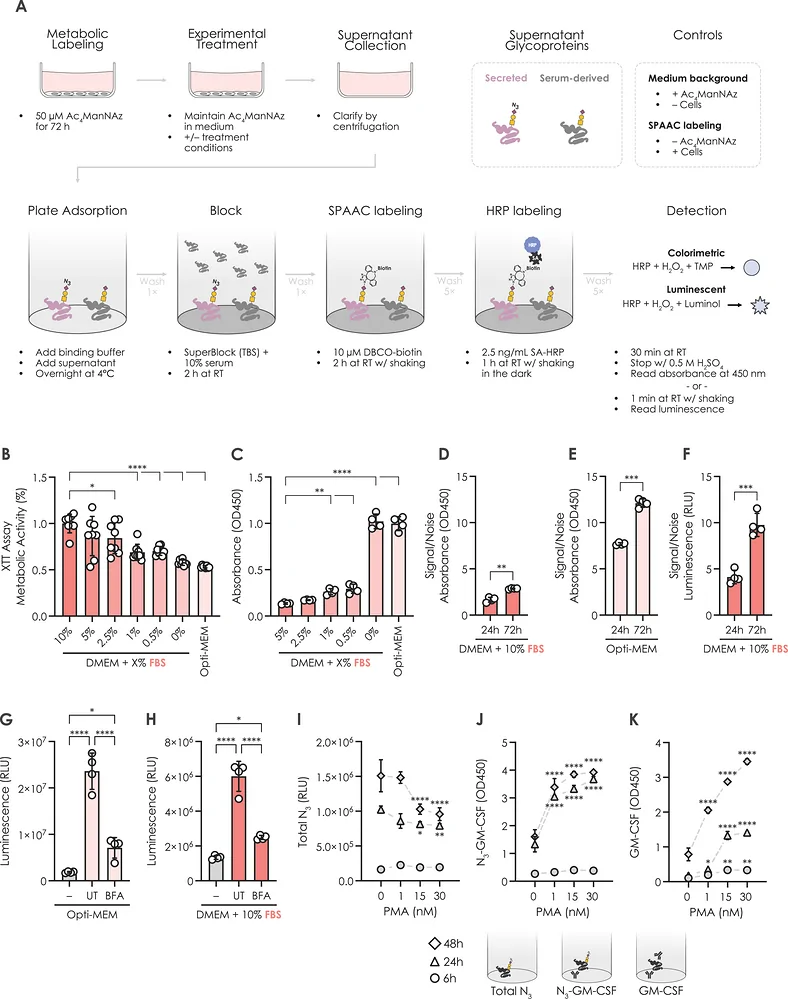
Quantifying secreted glycoproteins under standard culture conditions. (A) Schematic of the ELCA workflow. Supernatant samples containing azide‐modified glycoproteins are collected from metabolically labeled cells, adsorbed onto microwell plates, and subsequently labeled via strain‐promoted cycloaddition. Detection and quantification are performed using HRP. (B) XTT assay quantifying cellular metabolic activity under culture conditions with varying serum concentrations. (*n* = 8). (C) ELCA analysis of supernatants from cells cultured under varying serum concentrations using standard colorimetric detection (*n* = 4). (D, E) Signal‐to‐noise ratio (S/N) of ELCA signal comparing standard culture conditions (DMEM + 10% serum; (D) and serum‐free Opti‐MEM (E) at 24 h and 72 h time points, measured by standard colorimetric detection (*n* = 4). (F) S/N of ELCA signal from cells cultured under standard conditions at 24 h and 72 h time points, measured using high‐sensitivity luminescent detection (*n* = 4). (G, H) ELCA signal following treatment with 3 µm Brefeldin A (BFA) for 24 h under serum‐free (G) or standard culture conditions (H), measured using high‐sensitivity luminescent detection (*n* = 4). – (gray bars): blank control. (I–K) ELCA measurements of total glycoprotein secretion (I), Sandwich‐ELCA measurements of glycosylated GM‐CSF secretion (J), and ELISA measurements of total GM‐CSF secretion (K) following PMA stimulation in cells cultured under standard conditions for 6–48 h, using high‐sensitivity luminescent detection (I; *n* = 3) or standard colorimetric detection (J–K; *n* = 3). All assays were conducted in A375 cells. Statistical significance was assessed using a two‐tailed Welch's *t*‐test (two conditions), one‐way ANOVA with Dunnett's multiple comparisons test (>2 conditions, 1 variable), or two‐way ANOVA with Tukey's multiple comparisons test (<2 conditions, 2 variables). ^*^ *p* < 0.05, ^**^ *p* < 0.01, ^***^ *p* < 0.001, ^****^ *p* < 0.0001.

With the goal of quantifying secreted glycoproteins under standard culture conditions, including the presence of serum, we set out to optimize the ELCA workflow for maximal sensitivity. We found that careful titration of horseradish peroxidase (HRP), as well as the choice of blocking reagent and protocol, was critical for minimizing non‐specific background signal and achieving an optimal signal‐to‐noise ratio (S/N; Figure S1A,B). We note that these parameters may require validation and further optimization when switching reagent lots or establishing the assay in a new laboratory.

While serum starvation would reduce interference from highly abundant background proteins, we confirm that cellular metabolic activity is significantly affected under these conditions (Figure 1B), which risks introducing artificial changes into the secreted glycoproteome. Moreover, while short‐term serum starvation may be tolerated, the feasibility of time‐course experiments to monitor additional perturbations, e.g., small‐molecule treatments, would be limited. In addition, we evaluated the impact of Ac_4_ManNAz on cellular metabolic activity, observing no labeling‐dependent inhibition (Figure S1C). Tolerance to Ac_4_ManNAz is known to be cell line‐dependent [24], and it is therefore recommended to empirically determine the optimal metabolic labeling conditions to minimize any potential detrimental effects of the labeling itself.

Using standard colorimetric detection, we observed that the sensitivity of this first version of ELCA was significantly reduced in the presence of even small amounts of exogenous serum‐derived proteins (Figure 1C). This effect was likely due to interference with the capture of azido‐labeled proteins, as the assay relies on coating the microwell plate with the entire, complex sample, and serum proteins exceed labeled, cell‐derived material by orders of magnitude. Extending the culture duration from 24 h to 72 h provided small improvements, yet the overall signal‐to‐noise ratio and dynamic range remained low under serum‐containing conditions, compared to serum‐free conditions (Figure 1D,E).

To address this limitation, we evaluated alternative detection strategies and tested chemiluminescent substrates designed for ultra‐sensitive detection. Lowering the detection limit (170 fg/well vs 6 pg/well) substantially improved signal detection in both serum‐containing and serum‐free conditions (Figure 1F; Figure S1D). Using Brefeldin A (BFA) treatment, which effectively blocks secretion, we then observed a strong signal in control conditions that was reduced to near‐background levels upon treatment, both in serum‐containing and serum‐free supernatants (Figure 1G,H).

In parallel, we evaluated the feasibility of substituting the DBCO‐biotin/streptavidin‐HRP detection system for a directly DBCO‐conjugated fluorescent dye. This strategy, providing only a single reporter molecule per azide, rather than HRP‐mediated enzymatic signal amplification, resulted in lower overall sensitivity, with a specific, culture duration‐dependent signal only detected under serum‐free conditions (Figure S1E,F).

We confirmed that the detected signal is specific to sialylated glycoproteins upon metabolic labelling with Ac_4_ManNAz. Specifically, (i) no non‐specific labeling by DBCO occurred in the absence of metabolic labeling, (ii) no measurable signal was observed from medium containing Ac_4_ManNAz but no cells, and (iii) the signal was significantly reduced upon treating supernatants with a broad‐specificity sialidase prior to analysis, demonstrating that the Ac_4_ManNAz‐labeled glycoconjugates are sialidase‐sensitive (Figure S1G). We also evaluated metabolic labeling using Ac_4_GalNAz in a proof‐of‐concept experiment. Ac_4_GalNAz primarily labels GalNAc‐containing glycans (e.g., *O*‐GalNAc), but can also contribute to labeling of GlcNAc‐containing structures (including *O*‐GlcNAc and *N*‐glycans) through GALE‐mediated epimerization to GlcNAz [25,^26^]. Consistent with this, we detected a strong ELCA signal in supernatants from Ac_4_GalNAz‐treated cells that was insensitive to sialidase treatment (Figure S1G).

To further expand the potential applications of the method, we finally explored the quantification of a specific secreted glycoprotein, using GM‐CSF, a heavily glycosylated cytokine known to be induced in A375 cells upon PMA treatment [27], as an example. Using the standard ELCA workflow, we first observed a general decrease in total secreted glycoproteins upon PMA treatment across assayed concentrations and time points (Figure 1I). Interestingly, when applying an ELISA‐inspired sandwich setup, using a monoclonal antibody for capture followed by click‐based detection of azide‐labeled glycans, we detected a concentration‐dependent increase in glycosylated GM‐CSF (Figure 1J), demonstrating that our method can be adapted to monitor specific glycoproteins, in addition to global secretion profiles. Using a standard sandwich ELISA to quantify the total abundance of secreted GM‐CSF, we confirmed its PMA‐induced upregulation (Figure 1K). Importantly, while ELCA and sandwich‐ELCA measurements were sensitive to glycosylation inhibitor and sialidase treatments (Figure S1H,I,K,L), conventional ELISA measurements remained unaffected (Figure S1J).

### 2.2 ELCA Detects Changes in the Secreted Glycoproteome with High Temporal Resolution

The cell surface glycome reflects the state of a given cell. For example, dysregulated metabolic activity, such as during stress responses or cancer, has been shown to alter the composition of glycans presented to the extracellular environment. These changes can confer advantages to cancer cells by modulating interactions with immune cells, while also serving as potential biomarkers and therapeutic targets [8, 28].

Because measurable changes in the cell surface glycome depend on the turnover of existing glycoproteins, we hypothesized that the abundance or rate of glycoprotein secretion could provide a more sensitive readout for early detection of cellular perturbations. Consistent with this, brief treatment with cycloheximide (CHX) to inhibit protein translation did not result in detectable changes in the surface glycome within 24 h, as assessed by lectin staining (longer treatments were not feasible due to reduced cell viability) (Figure 2A,B). In contrast, analyzing the supernatant using ELCA revealed a clear reduction in newly secreted glycoproteins under the same conditions (Figure 2C).

**FIGURE 2:**
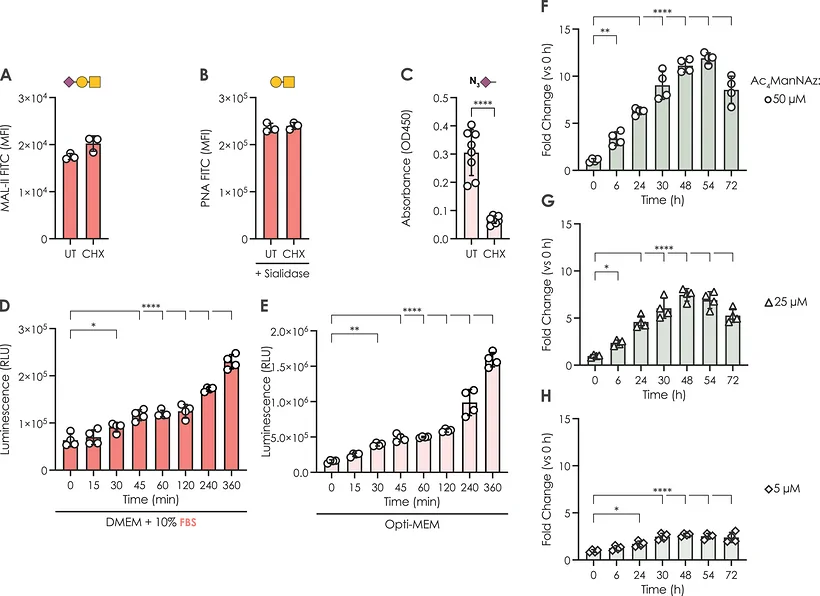
ELCA detects rapid changes in the secreted glycoproteome. (A, B) Lectin‐based flow cytometry analysis of cell surface glycoproteins using MAL‐II (A; Neu5Acα2‐3Galβ1‐3GalNAc) and PNA (B; Galβ1‐3GalNAc) lectin staining following inhibition of translation with 25 µm cycloheximide (CHX) for 24 h showed no significant changes (*n* = 3). (C) Under the same treatment conditions, a clear reduction in newly secreted sialoglycoproteins was detected using ELCA. Measurements were performed under serum‐free conditions using standard colorimetric detection (*n* = 8). (D, E) A375 cells were metabolically labeled with 50 µm Ac_4_ManNAz for 72 h, followed by re‐seeding in fresh serum‐containing DMEM (D) or serum‐free Opti‐MEM (E) medium. Supernatant samples were collected at the indicated time points and assayed with ELCA using high‐sensitivity luminescent detection (*n* = 4) (F, G). Serum‐containing DMEM medium supplemented with Ac_4_ManNAz (F: 50 µm; G: 25 µm; H: 5 µm) was added to A375 cells. At the indicated time points, the cells were then incubated in fresh serum‐free Opti‐MEM for 1 h, after which supernatants containing proteins secreted during this period were collected and stored at −20°C for ELCA analysis using high‐sensitivity luminescent detection (*n* = 4). Glycan structures have been drawn with GlycoDraw [29] and conform to the Symbol Nomenclature for Glycans [30] (SNFG). All assays were conducted in A375 cells. Statistical significance was assessed using a two‐tailed Welch's *t*‐test (two conditions) or one‐way ANOVA with Dunnett's multiple comparisons test (>2 conditions). ^*^ *p* < 0.05, ^**^ *p* < 0.01, ^****^ *p* < 0.0001.

To benchmark the sensitivity of ELCA, we next investigated the minimum secretion time required for reliable detection of glycoproteins in the supernatant. Under both standard culture conditions (Figure 2D) and serum‐free conditions (Figure 2E), a significant and specific signal was detectable after just 30 min of incubation with fresh medium, demonstrating the high sensitivity and temporal resolution of ELCA, even in the presence of highly abundant serum‐derived proteins.

Leveraging this sensitivity and ability to detect rapid changes, we next applied ELCA to monitor the dynamics of Ac_4_ManNAz metabolic labeling across different concentrations and time points. In A375 cells, we detected the secretion of azide‐tagged glycoproteins as early as six hours following the addition of Ac_4_ManNAz to the culture medium (Figure 2F,G). Furthermore, we were able to track labeling efficiency over time and observed time‐ and concentration‐dependent saturation with maximal labelling of newly secreted glycoproteins observed after around two days of incubation (48–54 h; Figure 2F–H). Interestingly, the observed secretion rate signal decreased again at 72 h (Figure 2F,G), highlighting the importance of considering azido‐sugar consumption and half‐life during longer experiments.

We then investigated whether the Ac_4_ManNAz concentration used for metabolic labeling affected the detection of treatment‐induced changes in glycoprotein secretion following exposure to Kifunensine or Brefeldin A. We observed strong linear correlations between measured ELCA signals in treated and control samples across Ac_4_ManNAz concentrations (Figure S2A), and while the relative treatment effects were most pronounced at 50 µm Ac_4_ManNAz, treatment‐induced changes remained robustly detectable even at substantially lower labeling concentrations (Figure S2B,C).

Given the suitability of ELCA for sensitive detection of glycoprotein secretion with high temporal resolution, we finally explored whether the temporal onset of measurable signal changes in response to a given perturbation could provide insight into its mechanism of action. Brefeldin A treatment, which disrupts trafficking at a late stage of the secretory pathway, produced measurable differences as early as 20 min after addition to the medium. In contrast, cycloheximide, which acts further upstream by inhibiting protein translation, resulted in detectable changes only after 120 min (Figure S3). Together, these observations suggest that the temporal onset of ELCA signal changes may reflect where a perturbation acts along the protein synthesis and secretory pathway.

### 2.3 ELCA Detects Small Molecule‐Induced Modulation of Glycosylation at Early Timepoints

Having demonstrated a temporal difference in the impact on cell surface versus secreted glycoproteins, we next turned to evaluating the dynamics of commonly used inhibitors of cellular glycosylation, including Benzyl‐α‐GalNAc (Benz; inhibiting *O*‐GalNAc extension) and Kifunensine (Kif; mannosidase I inhibition, causing accumulation of high‐mannose *N*‐glycans). To this end, we analyzed cell surface glycosylation by flow cytometry and secreted glycoproteins by ELCA in parallel from the same experimental samples (Figure 3A).

**FIGURE 3:**
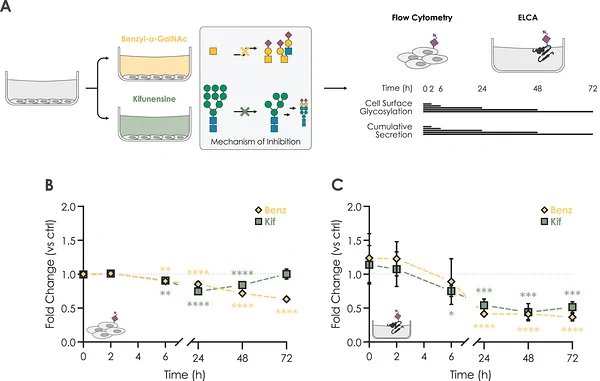
ELCA analysis of culture supernatant is superior to cell surface flow cytometry for detecting small molecule‐induced modulation of glycosylation. (A) Overview of the experimental setup. A375 cells were metabolically labeled with 50 µm Ac_4_ManNAz for 72 h, followed by re‐seeding in fresh serum‐containing medium supplemented with common glycosylation inhibitors (1 mm Benzyl‐α‐GalNAc, Benz, and 10 µm Kifunensine, Kif). At the indicated time points, supernatants were collected and stored at −20°C until ELCA analysis. Cells were washed once with Opti‐MEM, then incubated with 10 µm DBCO‐biotin for 1 h at 37°C and subsequently analyzed by flow cytometry. (B) Flow cytometry analysis of biotinylated cell surface glycoproteins using fluorescein‐conjugated streptavidin for detection (*n* = 4). (C) ELCA measurements of serum‐containing supernatants using high‐sensitivity luminescent detection (*n* = 4), reflecting cumulative protein secretion for the duration of the treatment. All assays were conducted in A375 cells. Ctrl: untreated control. Statistical significance was assessed using two‐way ANOVA with Tukey's multiple comparisons test (>2 conditions, 2 variables). ^*^ *p* < 0.05, ^**^ *p* < 0.01, ^***^ *p* < 0.001, ^****^ *p* < 0.0001.

Comparing the relative effects on cell surface and secreted glycoproteins in a time‐course experiment, we found that treatment with Benzyl‐α‐GalNAc induced a gradual, time‐dependent change in cell surface glycosylation. A significant but modest decrease was detectable after 6 h of treatment, reaching a maximal reduction of approximately 40% after 72 h (Figure 3B). In contrast, ELCA analysis of supernatant samples revealed more rapid and pronounced effects, with a sustained 60–75% reduction in signal observed throughout the 24–72 h time points (Figure 3C).

Analysis of cell surface glycosylation by flow cytometry showed that Kifunensine treatment resulted in a maximal signal reduction of approximately 25% after 24 h relative to the untreated control (Figure 3B), with signal levels returning to baseline by 72 h. In contrast, ELCA measurements of supernatant samples revealed a substantially greater effect, with signal reductions of up to ∼60% that persisted throughout the treatment period (Figure 3C). We thus caution researchers using these inhibitors to study membrane‐associated glycosylation, as sufficient timescales may be required to allow for equilibration in such experiments (e.g., Benzyl‐α‐GalNAc), while, at the same time, transient effects may be overlooked if sampling is performed outside the optimal detection window (e.g., Kifunensine).

Potential future applications of ELCA include high‐throughput screening for compounds that modulate glycoprotein secretion. We therefore evaluated the compatibility of the workflow with a 96‐well screening format. Using randomly assigned vehicle control and Kifunensine treatments across the plate, ELCA reliably distinguished Kifunensine‐treated wells from controls, demonstrating its potential suitability for screening applications (Figure S4A,B).

### 2.4 Quantifying Secreted Glycoproteins from THP‐1‐Derived Macrophages

To benchmark ELCA in a physiologically relevant model, we quantified secreted glycoproteins from THP‐1‐derived macrophages under resting state and during differentiation induced by polarizing treatments. Specifically, we compared naïve M0 macrophage‐like cells (PMA‐differentiated THP‐1), classically activated (M1) macrophages induced by LPS, and alternatively activated (M2) macrophages induced by IL‐4/IL‐13 (Figure 4A). These conditions are known to generate cell populations with distinct cytokine profiles and downstream effector functions.

**FIGURE 4:**
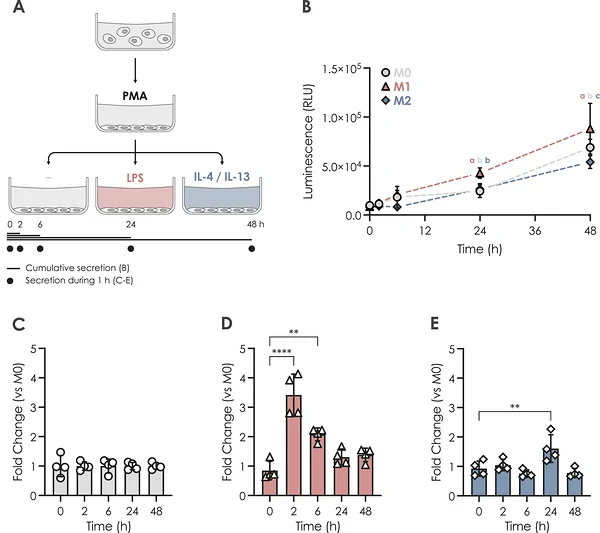
ELCA detects differential secretion profiles during THP‐1‐derived macrophage polarization. (A) Overview of the experimental setup. THP‐1 cells were differentiated into naïve macrophage‐like (M0) cells and metabolically labeled (20 nm PMA, 50 µm Ac_4_ManNAz for 72 h), followed by 24 h of rest and subsequent activation with LPS (100 ng/mL, M1) or IL‐4 and IL‐13 (20 ng/mL each, M2). At the indicated time points, serum‐containing supernatants were collected and stored at −20°C until ELCA analysis. Cells were then incubated in fresh serum‐free Opti‐MEM for 1 h, after which supernatants containing proteins secreted during this period were collected and stored at −20°C for ELCA analysis. (B) ELCA measurements of serum‐containing supernatants using high‐sensitivity luminescent detection (*n* = 4), reflecting cumulative protein secretion for the duration of the treatment. At each time point, letters indicate statistical groupings. (C–E) ELCA measurements of serum‐free supernatants using high‐sensitivity luminescent detection (*n* = 4), reflecting the abundance of proteins secreted during the 1 h incubation period. All assays were conducted in THP‐1 cells. Statistical significance was assessed using one‐way ANOVA with Dunnett's multiple comparisons test (>2 conditions, 1 variable) or two‐way ANOVA with Tukey's multiple comparisons test (>2 conditions, 2 variables). ^**^ *p* < 0.01, ^****^ *p* < 0.0001.

Measurements of cumulative secretion at the indicated time points throughout the experiment revealed a significantly higher abundance of secreted glycoproteins in LPS‐activated M1 macrophages from 24 h onward (Figure 4B). At each time point, we also measured secretion over a 1 h period to assess secretion rates across conditions (Figure 4C–E). In strong agreement with previous reports on human macrophage activation [31], we observed a strong, early response to M1‐polarizing treatment (Figure 4D), whereas M2‐polarizing treatment resulted in a more gradual and delayed response (Figure 4E).

## 3 Discussion

ELCA offers a flexible approach for detecting and quantifying secreted glycoproteins. Cells can be monitored under physiology‐mimicking growth conditions, i.e., including the presence of high‐abundance FBS‐derived (glyco)proteins, while maintaining sufficient sensitivity to detect rapid, small‐molecule‐induced changes in glycosylation and/or glycoprotein abundance.

Detection sensitivity can be further enhanced by using serum‐free conditions in experiments where this is tolerated. In addition, cells may first be cultured under standard conditions, preserving viability during long‐term treatments, to measure cumulative secretion over the course of the experiment. Then, at the experimental endpoint, secretion rates can be estimated by quantifying proteins secreted into fresh serum‐free medium during a defined short‐term interval, such as 1 h.

Bioorthogonal metabolic labeling with Ac_4_ManNAz enables the detection and quantification of global sialoglycoproteins. By using alternative azide‐functionalized monosaccharide precursors, the method can be adapted to quantify specific glycan classes, as demonstrated here with Ac_4_GalNAz for *O*‐GalNAc glycoproteins. Although additional optimization and validation would be required, the approach should also be readily adaptable to an ever‐growing plethora of selective bioorthogonal probes, including azide‐functionalized fucose analogs for the detection of fucosylated glycans [32] or 1,3‐Pr_2_‐6‐OTs GlcNAlk for hybrid *N*‐glycan structures [33] (using azide‐biotin for detection in the latter case).

Analogous to how exoglycosidase treatment is being used to endow mass spectrometry with higher structural resolution [34], ELCA could potentially be further customized by pre‐treating samples with, e.g., α2‐3 linkage‐specific sialidases prior to the click reaction to only quantify α2‐6 linked sialylation, if needed for a biological question.

While alternative methods can provide detailed structural insights into glycans, ELCA is well suited for initial screening and high‐throughput experimental designs involving many replicates and/or conditions. We also envision ELCA to find use in screening new glycosylation inhibitors that are needed to advance glycobiology. With an approximate cost of 1–2 EUR per sample (assayed in technical duplicates in a 96‐well plate format) for standard colorimetric or high‐sensitivity luminescent detection, respectively, ELCA is orders of magnitude less expensive than dedicated glycomics or glycoproteomics approaches. At the same time, it remains highly accessible, as it does not require specialized equipment.

ELCA signal is proportional to the number of accessible azide‐modified sialic acids adsorbed per well, rather than to the molar amount of protein, so multiply sialylated proteins contribute more signal per molecule. Therefore, an observed change in ELCA signal may reflect changes in the total amount of secreted glycoproteins and/or the degree of glycosylation per protein, with further validation using orthogonal methods required to distinguish these possibilities. Glycosylation per molecule of specific proteins can be quantified using the ELISA‐inspired, monoclonal antibody‐based capture variant of ELCA we present here, in combination with normalization to protein abundance as measured by conventional methods, such as standard ELISA or Western blotting.

While the current application of our method is presented with a focus on glycoprotein quantification, we caution that a glycolipid‐derived contribution to the signal cannot be excluded. Small extracellular vesicles are efficiently labeled by Ac_4_ManNAz [35] and, although cell line‐dependent, can be enriched in gangliosides [36]. Demonstrating the potential contribution of these vesicles to the ELCA signal will require future dedicated fractionation studies.

Metabolic labeling unavoidably increases the intracellular levels of sialic acid substrate available for sialylation [24], potentially impacting overall glycosylation abundance and patterns [37]. Importantly, Ac_4_ManNAz is present at identical concentration across experimental and control conditions here, so any substrate‐driven baseline shift is shared across the comparison. Thus, ELCA reports relative differences between matched conditions under a constant precursor load, not absolute sialylation. Further, it is known that the usage of azide‐containing sialic acid may shift the distribution of sialyltransferase activity in subtle ways [38]. As standard ELCA merely reports on global sialylation levels, regardless of linkage, we deem this of lesser concern for most applications.

## 4 Methods

### 4.1 Mammalian Cell Culture

A375 cells were cultured in Dulbecco's Modified Eagle Medium (DMEM) with high glucose, 4.0 mm L‐glutamine, and sodium pyruvate (SH30243.01, Cytiva). THP‐1 cells (TIB‐202, ATCC) were cultured in Roswell Park Memorial Institute (RPMI) 1640 medium (ATCC modification; A1049101, Gibco). The respective growth media were supplemented with 10% (v/v) heat‐inactivated fetal bovine serum (FBS; FBS‐HI‐12A, Capricorn Scientific) and 1% (v/v) penicillin‐streptomycin solution (P4333‐100ML, Sigma‐Aldrich). All cells were maintained at 37°C in a humidified incubator with 5% CO2.

THP‐1 cells were differentiated into macrophage‐like cells by treatment with 20 nm phorbol‐12‐myristate‐13‐acetate (PMA; P8139‐1MG, Sigma‐Aldrich) for 72 h followed by a 24 h rest period in fresh medium. Classically activated (M1) macrophages were induced by treatment with 100 ng/mL lipopolysaccharide (LPS; L4391‐1MG, Sigma‐Aldrich) and alternatively activated (M2) macrophages were induced by treatment with 20 ng/mL interleukin 4 (IL‐4; SRP3093‐20UG, Sigma‐Aldrich) and 20 ng/mL interleukin 13 (IL‐13; SRP3274‐10UG, Sigma‐Aldrich).

Unless stated otherwise, metabolic labeling was performed by growing the cells in the presence of 50 µm Ac_4_ManNAz (CLK‐1084‐25, Jena Bioscience) for 72 h. Small molecule‐mediated modulation of cellular glycosylation was performed by treatments with 1 mm Benzyl‐α‐GalNAc (HY‐129389, MedChemExpress) and 10 µM Kifunensine (HY‐19332, MedChemExpress). For all treatments, the untreated control included matched concentrations of vehicle solvent.

### 4.2 Enzyme‐Linked Cycloaddition Assay

A step‐by‐step protocol is available in Supplementary Methods. Briefly, A375 cells were metabolically labeled with Ac_4_ManNAz prior to seeding in 24‐well plates (500 µL per well, 0.25 × 10^6^ cells/mL). Supernatants were collected and stored at −20°C until further analysis. For ELCA, 50 µL of supernatant was mixed with 50 µL of 0.2 m carbonate–bicarbonate pH 9.4 buffer (28382, Thermo Fisher) and adsorbed onto 96‐well MaxiSorp flat‐bottom plates (clear or white plates for colorimetric or luminescent detection, respectively) overnight at 4°C. Blocking was performed using SuperBlock (TBS) Blocking Buffer (37535, Thermo Fisher) supplemented with 10% FBS for 2 h at room temperature. Strain‐promoted azide‐alkyne cycloaddition was carried out by incubating with 10 µm DBCO‐PEG4‐Biotin (HY‐130809, MedChemExpress) or PC DBCO Biotin (CCT‐1120‐1, Vector Laboratories) in SuperBlock (TBS) Blocking Buffer for 2 h at room temperature with gentle shaking (500 rpm). HRP labeling was performed by incubation with 2.5 ng/mL streptavidin‐HRP (N100, Thermo Fisher) in SuperBlock (TBS) Blocking Buffer for 1 h at room temperature with gentle shaking. Detection was carried out using either a colorimetric substrate (TMB Substrate Kit; 34021, Thermo Fisher) or a luminescent substrate (SuperSignal ELISA Femto Substrate; 37075, Thermo Fisher), according to the manufacturer's instructions. Washing steps consisted of five washes with TBS‐T, each with a 1 min incubation and gentle shaking. Sialidase treatments were performed using 0.05U α2‐3/6/8‐Sialidase (10269611001, Sigma‐Aldrich) in 50 mm sodium acetate pH 5.2 (S7899, Sigma‐Aldrich) for 2 h at room temperature.

### 4.3 GM‐CSF Quantification

For the detection of glycosylated GM‐CSF, ELCA was combined with a standard sandwich ELISA‐based antigen capture approach. Briefly, clear 96‐well MaxiSorp flat‐bottom plates were coated overnight at 4°C with 1 µg/mL GM‐CSF monoclonal antibody (clone 3092; M500A, Thermo Fisher) and subsequently blocked with 3% BSA for 1 h at room temperature. 100 µL supernatant was added per well, incubated for 2 h at room temperature for capture, and then click‐reacted with 10 µm PC DBCO Biotin (CCT‐1120‐1, Vector Laboratories) for 2 h at room temperature. Streptavidin‐HRP labeling and colorimetric detection were performed as described above.

Total GM‐CSF was quantified using an in‐house sandwich ELISA setup. Briefly, clear 96‐well MaxiSorp flat‐bottom plates were coated overnight at 4°C with 1 µg/mL GM‐CSF monoclonal antibody (clone 3092; M500A, Thermo Fisher) and subsequently blocked with 3% BSA for 1 h at room temperature. 100 µL supernatant was then added per well, and the plate was incubated for 2 h at room temperature. For detection, the plate was incubated with 0.5 µg/mL GM‐CSF polyclonal antibody (17762‐1‐AP, Proteintech) for 1 h at room temperature followed by 40 ng/mL HRP‐conjugated Goat Anti‐Rabbit IgG (SA00001‐2, Proteintech) for 1 h at room temperature. Colorimetric detection was carried out as previously described.

### 4.4 XTT Assay

20 000 cells (100 µL of 0.2 × 10^6^ cells/mL) were seeded in each well of a 96‐well plate. After incubation for 24 h under the assayed experimental conditions, 50 µL XTT labeling mixture (5 mL XTT labeling reagent + 0.1 mL electron coupling reagent for one full 96‐well plate) was added, and the plate was incubated for 4 h. Background absorbance measured at the reference wavelength 660 nm was subtracted from the specific absorbance of the formazan product measured at 450 nm. The obtained values were normalized to standard culture conditions (DMEM + 10% FBS) and a blank medium control without cells and are reported as metabolic activity (%).

### 4.5 Flow Cytometry Detection of Azide‐Tagged Cell Surface Glycoproteins

The cells were washed once in Opti‐MEM (31985070, Thermo Fisher) and then incubated with 10 µm DBCO‐PEG4‐Biotin (HY‐130809, MedChemExpress) or PC DBCO Biotin (CCT‐1120‐1, Vector Laboratories) in Opti‐MEM for 1 h at 37°C with 5% CO_2_. After labeling, the cells were washed in normal culture medium for 1 h before harvesting. Detached cells were washed once in PBS and then incubated with 2 µg/mL streptavidin‐fluorescein (SA‐5001‐1, Vector Laboratories) for 30 min at 4°C. Surface staining was done together with the LIVE/DEAD Fixable Far Red Dead Cell Stain Kit (L10120, Thermo Fisher, 1000× dilution). Data were acquired on an Accuri C6 Plus Flow Cytometer (BD) and analyzed using FlowJo 10.10.0 software.

### 4.6 Quantification and Statistical Analysis

Comparing two groups was done via two‐tailed Welch's *t*‐tests. Comparing more than two groups was done via one‐way ANOVA with Dunnett's multiple comparison test (1 variable) or via two‐way ANOVA with Tukey's multiple comparisons test (2 variables).

## Author Contributions

D. B. and J. L. conceptualization; J. L. and J. Y. formal analysis; D. B., J. L., and J. Y. writing – original draft; D. B., J. L., and J. Y. writing – review & editing; J. L. visualization; D. B. supervision; D. B. funding acquisition; D. B., J. L., and J. Y. methodology.

## Conflicts of Interest

D. B. is consulting on glycobiology‐related topics via SweetSense Analytics AB. The remaining authors declare no competing interests.

## Supporting information




**Supporting File**: advs78315‐sup‐0001‐SuppMat.pdf.

## Data Availability

The data that support the findings of this study are available in the supplementary material of this article.
